# Valorization and Environmental Impacts of Pecan Waste: A Critical Review

**DOI:** 10.3390/foods15010168

**Published:** 2026-01-04

**Authors:** Jean Louis Yannick Omotonoko, Michael Polozola, Andrej Svyantek, Zhuoyu Wang

**Affiliations:** 1 Water Management & Hydrological Science Department, Texas A&M University, College Station, TX 77843, USA; 2 Food Sciences & Technology Department, Texas A&M University, College Station, TX 77843, USA; 3 Dean Lee Research Station, Louisiana State University, Alexandria, LA 71302, USA; 4 Department of Horticultural Sciences, Texas A&M University, College Station, TX 77843, USA

**Keywords:** pecan shell, valorization, circular economy

## Abstract

Pecan (*Carya illinoinensis*) cultivation generates a substantial number of byproducts, particularly nutshells, which are often discarded despite being rich in bioactive and structural compounds. These agro-industrial residues, comprising nearly 50% of the total nut mass, contain high levels of phenolics, flavonoids, dietary fiber, and lignocellulosic matter, making them suitable for circular economy applications. This review critically evaluates the potential of pecan shell waste for value-added applications in environmental remediation, food and pharmaceutical formulations, and green materials production. It explores innovative green extraction techniques, such as ultrasound-assisted, microwave-assisted, and subcritical water extraction, to recover valuable compounds like ellagic acid and tannins with high efficiency and minimal environmental impact. Moreover, the review highlights the conversion of pecan shells into activated carbon for wastewater treatment and soil remediation. Pecan byproducts have been used as sustainable feedstocks for catalyst support, contributing to energy conversion and biomass catalysis. The bioactive compounds also offer therapeutic properties, including antioxidant, anti-inflammatory, and antimicrobial effects, supporting their inclusion in nutraceutical and cosmetic applications. Through a comprehensive synthesis of recent studies, this work highlights the role of pecan shell valorization in reducing waste, improving public health, and increasing economic resilience within agro-industrial systems. By aligning with sustainable development and circular economies, the utilization of pecan byproducts provides a low-cost, eco-innovative pathway to mitigate environmental pollution and promote sustainable development.

## 1. Introduction

Current global population growth is leading to an increasing demand for food production [1]. The global agroindustry produces billions of metric tons of waste annually, much of which remains underutilized, posing significant challenges to environmental sustainability [2]. In 2021–2022, global pecan nut (*Carya illinoinensis*) production was approximately 129,510 metric tons [3]. The United States and Mexico were the leading producers, accounting for approximately 45% and 39% of the total output. South Africa is the third-largest pecan producer and has likely continued to increase pecan production. Argentina, although a more minor player, produced around 2000 tons from 10,000 hectares in 2021 and is projected to expand its cultivation area by 700 to 900 hectares annually [4]. Notably, South Africa and Argentina are located in the Southern Hemisphere, where the pecan harvest typically occurs from April to June, which differs from the Northern Hemisphere’s season, which spans from October to December. This geographic spread may help support more continuous global availability of pecan shell byproducts for industrial and research applications. While pecan nuts are increasingly cultivated worldwide for their nutritional benefits, the disposal of large volumes of nutshell waste remains problematic due to their slow degradation rate and minimal incorporation into circular economy systems [4]. Pecan shells constitute approximately 40–50% of the total nut mass; however, in native and low-input systems, the shell percentage may range to 55%. The variability may influence the volume and characteristics of shell biomass available for valorization [5]. They are rich in phenolic compounds, mono- and polyunsaturated fatty acids, phytosterols, tocopherols, and essential micronutrient components known to contribute to the prevention of various chronic diseases [6]. This underutilized biomass represents a missed opportunity to develop sustainable applications that align with the principles of green chemistry and waste prevention [6]. Pecan nutshells have attracted increasing interest as a source of bioactive compounds, and higher concentrations of phenolics and flavonoids have been identified in the shells compared to the edible nut [1]. Various strategies have been developed to valorize agricultural residues, converting them into high-value-added products, such as biofuels, nutraceuticals, and sustainable materials [5]. These efforts often align with circular economy principles, which emphasize reducing waste and maximizing resource reuse, and increasingly rely on green extraction techniques that lower energy consumption and avoid using toxic solvents. The versatility of these emerging technologies highlights the largely unexplored potential of pecan processing residues as a sustainable source of natural antioxidants and bioactive compounds, with promising applications in the food, pharmaceutical, and cosmetic industries. Recent research highlights the potential of agricultural residues, such as pecan shells, to be transformed into value-added products, including activated carbon for environmental remediation and soil amendments for agricultural use [7]. However, their widespread application remains limited without a comprehensive understanding of their long-term impacts on soil health, nutrient dynamics, and environmental risks. It is essential to investigate the physicochemical effects on soil systems to ensure safe and effective integration of pecan-based waste into sustainable agricultural practices. Developing evidence-based application rate and timing guidelines under varying conditions will guide pecan nutshell utilization while avoiding unintended environmental consequences. This review aims to explore the ecological impacts of pecan nutshell waste and examine current and emerging strategies for its valorization, particularly in the context of sustainable development goals, resource management, and circular economy principles.

## 2. Methods

**Figure 1:**
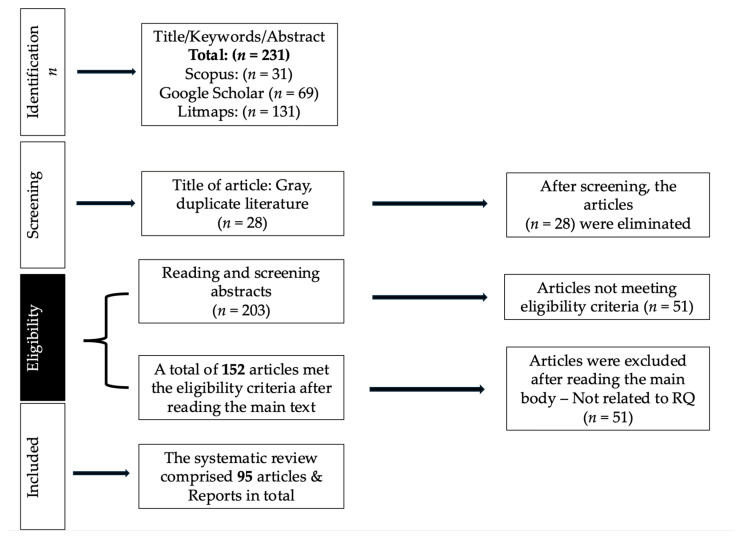
PRISMA-based workflow of study selection in the systematic review.

This review followed a systematic literature review approach (Table 1) aligned with the PRISMA framework (Figure 1). A total of 231 entries were initially identified from Scopus (*n* = 31), Google Scholar (*n* = 69), and Litmaps (*n* = 131). After deleting 28 duplicates and grey literature, 203 articles underwent title and abstract screening. Of these, 152 papers fulfilled the preliminary eligibility criteria and underwent full-text review. Following a thorough review, 66 papers were excluded for failing to meet the study topics, and 51 were deleted for poor methodological relevance or incomplete data. Ultimately, 86 studies met all inclusion criteria and were chosen for the final systematic analysis. Extraction processes, physicochemical properties, value chain paths, and environmental uses of pecan shell-derived compounds were among the variables extracted.

## 3. Pecan Shells and Utilizations

### 3.1. Pecan Growth Areas and Other Basic Growth Conditions

Native to areas along the Mississippi River, pecans thrive in deep, fertile, and well-drained soils. Its natural habitat extends from Mexico to southern Illinois in the United States, with trees thriving in warm temperate and subtropical temperatures [8]. To optimize pecan growth, several environmental conditions must be met. Warm and long summer is preferred for pecan growth. Pecan tree growth is restricted when soil temperature falls below 18°, making deep, fertile, and well-drained soils crucial for optimal pecan growth and development [8]. Pecan trees require substantial irrigation, particularly during peak growth and nut development. In mature orchards, individual trees may need between 150 and 250 gallons of water per day, depending on the climate and soil conditions, whereas for common tree crops, such as apples, only 1–10 gallons of water is required [9,10]. On an orchard scale, drip and micro-irrigation systems are typically designed to deliver 3600 to 4000 gallons per acre per day. Comparisons among the nut trees show that the water use efficiency in pecan trees is low (about 14–30 kg nuts ha^−1^ cm^−1^) due to their high-water demand [10]. These figures vary by region, cultivar, and management intensity, but they underscore the water demands of pecan production and the importance of efficient irrigation strategies. Intense irrigation can potentially impact water resources and soil composition [11]. First-year plants start with a deep taproot, and in the following years, lateral roots and vertical absorptive root formation follow [4]. Fibrous roots and thick mycorrhizal networks greatly aid nutrient acquisition, particularly in the upper soil layers. While higher-elevation sites or gentle slopes are often recommended to reduce cold damage and improve drainage, particularly in regions prone to spring frosts or heavy rainfall, many commercial orchards, especially in the southern United States and other flatland production zones, are successfully managed on level terrain. In these settings, growers rely on cultivar selection, orchard spacing, and canopy management to mitigate disease pressure and optimize growing conditions [12].

### 3.2. Current Issues and Pecan Shell Composition

Pecan shells, a byproduct of the pecan nut industry, present both environmental challenges and opportunities for valorization. As with many agricultural residues, the improper disposal of pecan shells can contribute to environmental degradation, including soil and water contamination. Pesticide utilization is another concern related to the disposal or reutilization of pecan shells [13]. However, their rich chemical composition and physical characteristics make them a promising resource for value-added applications [14].

**Figure 2:**
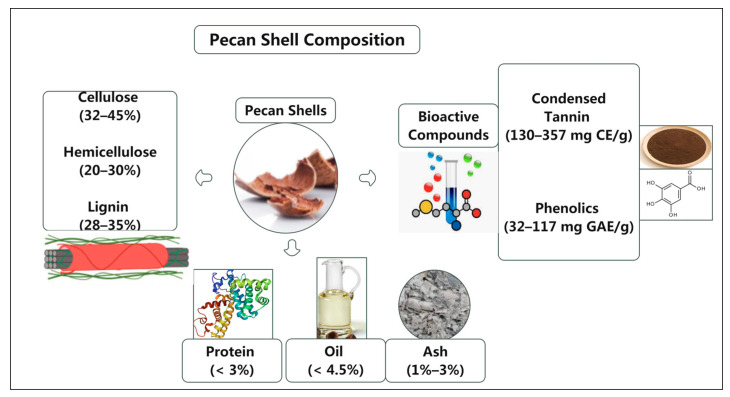
Pecan shell composition. The Unit CE represents catechin equivalents; GAE represents gallic acid equivalents.

Pecan shells primarily comprise cellulose, lignin, and hemicellulose, which constitute the majority of their lignocellulosic fibers, with cellulose representing about 32–45%, hemicellulose 20–30%, and lignin 28–35% of the total biomass (Figure 2). Pecan shells also contain less than 3% protein, less than 4.5% oil, and less than 3% ash content [15]. Chemically, pecan shells are composed of bioactive compounds, including tannins and phenolic substances, which are known for their potent antioxidant properties [14]. This complex composition makes them suitable for various valorization processes, such as the production of biofuels, biochemicals, and bio-based materials. The physicochemical makeup of pecan shells raises critical environmental considerations and positions them as a valuable resource for sustainable material and energy solutions. Shell thickness and density vary among pecan cultivars, influencing the column and physical characteristics of shell biomass available for valorization. Native and low-input cultivars often produce nuts with thicker shells. Sometimes the shells account for more than 55% of the total nut mass, whereas the improved commercial cultivars tend to have thinner shells optimized for kernel yield. These differences affect the suitability of shells for specific applications. Thicker shells may be more advantageous for biochar and activated carbon production due to their higher lignin and carbon content. In comparison, thinner shells may yield higher extractable phenolics for nutraceutical usage. Understanding cultivar-specific shell trains can help tailor processing strategies and improve efficiency in pecan waste utilization. Pecan shells from different cultivars differed primarily in thickness, which further affected texture. A higher kernel percentage often indicates a thinner shell relative to the nut size. Native pecans are usually smaller with thicker shells, which would provide more shell valorization material. American improved pecan varieties often have larger nuts but thinner shells. The compounds in pecan shells can vary and play an essential role in pathogen defense [16].

### 3.3. Pecan Waste Valorization

The valorization of agricultural residues has emerged as a critical area of focus, employing physical, chemical, and biochemical processes to generate feeds, fuels, energy, fiber-based materials, and a wide range of high-value chemical products [5]. Technologies used for the valorization of pecan shell residues include pyrolysis and gasification for biochar and bio-oil production, hydrothermal carbonization for carbon-rich solids, and solvent-based extractions (including ethanol, supercritical CO_2_, and ultrasound-assisted extraction) to recover polyphenols and other bioactive compounds [17]. Mechanical grinding and defatting are used as pre-treatment steps, followed by green extraction techniques such as microwave-assisted extraction and enzyme-assisted hydrolysis for use in nutraceuticals, pharmaceuticals, and cosmetics [3]. The versatility of these emerging technologies underscores the largely unexplored potential of pecan processing waste as a sustainable source of natural antioxidants and bioactive compounds, with promising applications in the food, pharmaceutical, and cosmetic industries. By transforming this agricultural byproduct into diverse, high-value-added products, pecan shells offer a scalable, waste-reducing solution that supports global environmental sustainability goals.

### 3.4. Alignment with the United Nations Sustainable Development Goals

Pecan shell valorization aligns well with the United Nations Sustainable Development Goals for 2025, which focus on ending poverty, protecting the planet, and ensuring prosperity for all by 2023, especially for Goals 12 and 13 [18]. Goal 12 aims to decouple economic growth from environmental degradation by using resources efficiently, reducing waste, promoting green practices, and encouraging businesses to adopt sustainable models, ultimately protecting the planet for future generations. Pecan shell, a common pecan processing waste, if used wisely, could significantly contribute to sustainable goods and businesses, which, in the long term, can promote consumers’ lifestyles and awareness. All subsequent applications, from food products to materials, from environmental amendments to energy production, could contribute to sustainable goals. Meanwhile, pecan shell valorization is one strategy to adapt to climate change, which is Goal 13. To strengthen resilience and adaptive capacity to climatic disasters, sustainable products derived from large amounts of agricultural waste and food byproducts have become the optimal choices to increase capacity to meet climate change.

### 3.5. Bio-Energy Production Applications

**Figure 3:**
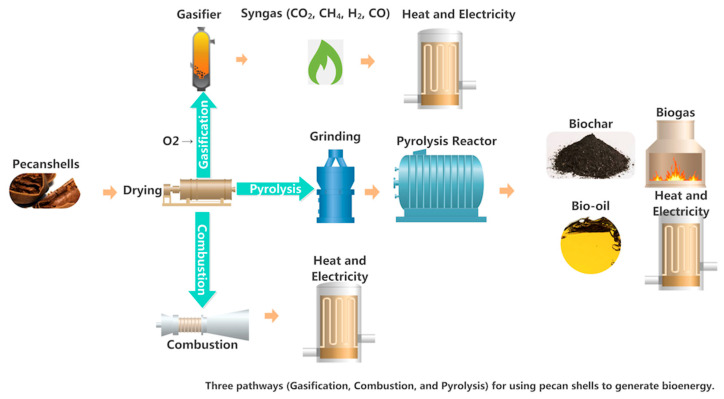
Bio-energy production with pecan shells.

Pecan nutshells, a plentiful agro-industrial byproduct, have become a significant lignocellulosic feedstock for bioenergy generation [19]. Their high carbon content, minimal ash, and high energy density render them suitable for various thermochemical and biochemical conversion processes designed to provide renewable energy and mitigate waste management [20]. By utilizing non-isothermal thermogravimetric analysis and pyrolysis-gas chromatography/mass spectrometry (Py-GC/MS), previous studies elucidated that the thermal decomposition of pecan shells transpires through three concurrent devolatilization reactions: hemicellulose (DE-HC), cellulose (DE-CL), and lignin (DE-LG) with different degradation temperatures [20]. Three process yields were generated: biochar (solid fraction), non-condensable gases (CO_2_), and condensable vapors (phenols, acids, furans, and hydrocarbons). These items can also be further utilized to generate energy, enhance soil quality, and produce bio-based compounds. Based on their heating values and fixed carbon content, pecan shells are a better biomass fuel than sugarcane bagasse or rice husk. The pecan ash content made it suitable for the combustion system, with reduced slagging during combustion, which benefits heat transfer and combustion efficiency [7]. For bioenergy conversion, pecan shells can be utilized through direct combustion, pyrolysis, and gasification (Figure 3). Combustion is the quickest method to release the energy from pecan shells, which involves an oxidizer (e.g., oxygen). However, this method primarily relies on fuel in the combustion process, which can cause water, noise, and air pollution [21]. Gasification is a thermochemical process that could convert pecan shells into a gaseous product called synthesis gas (syngas). The controlled amount of oxygen will react with the pecan shells as the carbonaceous substance. Due to the high heat and oxygen, the cost will be higher than combustion. Greenhouse gas emissions and water use are key environmental concerns associated with this technology [22]. Pyrolysis is the most efficient method, producing the highest energy output and generating bio-oil, syngas, char, and other fuels. Therefore, it can play a role in creating a circular economy by transforming pecan shells into energy. Since the procedure is more complex, the initial investment cost is higher than other methods, such as combustion or gasification; however, its long-term benefits, leading to product diversity and environmental sustainability, are significant. Technologies such as fluidized bed reactors, microwave-assisted pyrolysis, and co-pyrolysis can be considered for further improvement in utilizing pecan as a bioenergy resource. Additionally, optimizing process parameters, including temperature, heating rate, and residence time, alongside pre-treatment and strategic feedstock blending, is crucial to maximizing biochar output and economic viability [22]. By incorporating these technological advances, the valorization of pecan shells can become a key component of sustainable waste management and renewable energy generation systems.

Environmentally, the bioenergy derived from pecan shells offers climate benefits without posing risks of deforestation, biodiversity loss, or conflicts over water and food security, since pecan shells are the typical by-products of pecan production. Pecan shell activated carbon can also be further modified through chemical, organic/inorganic loading, physical, and other methods. The modified activated carbon provides high adsorption capacity and can be used to remove contaminants such as heavy metals and chemical residues [23].

However, pecan shells, like other biofuels, are not always nutrient neutral, releasing greenhouse gases or pollutants [24]. Still, compared to fossil fuels, agricultural waste usually reduces greenhouse gas and pollutant emissions, without accounting for land use change. For example, biomass energy could increase ethanol combustion, further reducing carbon monoxide emissions. Compared to fossil fuels, the potential for biomass production can mitigate ecosystem loss, especially in soil systems. Meanwhile, the use of pecan shells can reduce land and water use for common bioenergy crops, such as corn, soybeans, and silver grass [25].

### 3.6. Bioplastics and Other Sustainable Materials

**Figure 4:**
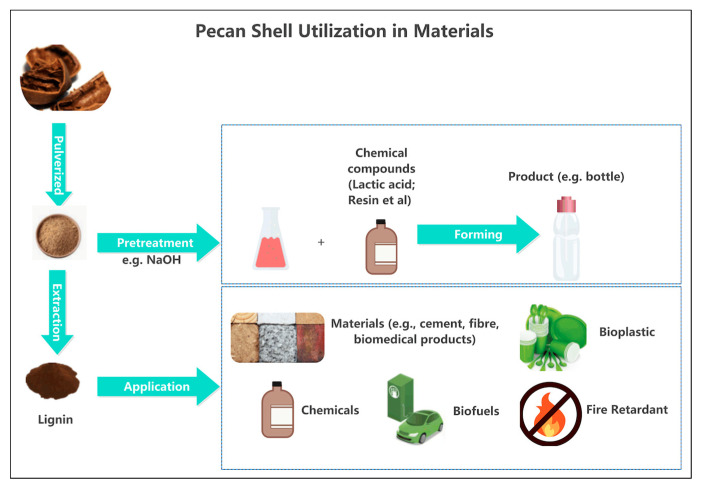
Pecan shell utilization in materials.

Pecan shells have promise as a source for sustainable material valorization due to their high lignocellulosic content [1]. Lignin can be extracted from pecan shells and utilized in industry, biomedicine, and agroindustry [26]. Pecans produce a significant amount of shell waste that can be used to regenerate materials, such as biodegradable plastics, replacing traditional oil-based plastics. Research on alkali-treated walnut shells has shown that surface modification with NaOH enhances their compatibility with bio-based polymers such as polylactic acid (PLA) and generates biopolymers with increased strength and thermal stability [27]. This model could be applied to pecan shells. Non-cellulosic components in pecan shells can be removed by alkali or enzymatic pretreatment, thereby increasing the material’s crystallinity and matrix adherence when used in bioplastic or composite manufacturing. The qualities of cellulose and lignin can be enhanced by additional procedures, such as mechanical dispersion or bleaching, making them suitable for bioplastics, composites, and sustainable materials (Figure 4). Lignin can be extracted from biomass through various techniques, such as organic solvent, alkaline, or enzymatic pretreatment methods. These help preserve its functional integrity and enable its application in catalyst supports, carbon materials, and biopolymer composites. Recent studies have emphasized the potential of tailored lignin structures for high-value applications, including metal catalyst carriers, biosorbents, and sustainable fillers in green materials [28]. Pecan nutshell, as a biofiller, has been used in polylactic acid materials for rigid packaging and other applications [29]. The reinforcing filler in biodegradable polymers is a cost-effective and eco-friendly material [30]. The integration of pecan shells into such goods promotes circular economy concepts and transforms agro-industrial waste into sustainable, low-cost raw materials. This strategy helps minimize agricultural residue pollution and contributes to the creation of environmentally friendly industrial materials that replace fossil-based inputs, promoting green manufacturing and sustainability.

### 3.7. Pecan Shells for Functional Foods

Representing up to 50% of the total nut mass, pecan shells are rich in phenolic compounds, flavonoids, tannins, and fiber, which contribute to their antioxidant, antimicrobial, anti-inflammatory, and potentially anticancer properties [5]. These functional properties make pecan shells the ideal ingredients for nutraceutical products (antioxidants, fiber, colorants) and natural preservatives (Table 2).

Many types of phenolic acids, flavonoids, and proanthocyanidins have been identified in pecan nutshells (Table 2). As one of the main byproducts of pecan, pecan shells extract contains many bioactive compounds, such as myricetin, ellagitannins, and catechins, which offer various health benefits [59]. Among the primary bioactive compounds identified in pecan shells are ellagitannins, ellagic acid, catechins, myricetin, quercetin, gallic acid, and tannins, along with a high content of dietary fiber [60]. Ellagitannins and ellagic acid, abundant in pecan shells, exhibit potent antioxidant activities due to their high phenolic content [61]. These compounds contribute to health benefits by neutralizing free radicals and may also exert anti-inflammatory and anticancer effects. The antioxidant capacity is primarily attributed to their ability to scavenge reactive oxygen species and inhibit lipid peroxidation. Moreover, ellagic acid is recognized for its role in promoting health through the gut microbiota’s conversion into urolithins, thereby enhancing bioavailability and extending its biological effects [62]. Catechins help improve cardiovascular health, reduce oxidative stress, and support neurocognitive function [63]. Myricetin and quercetin exhibit anti-diabetic, anti-inflammatory, and immune-modulatory properties, with additional roles in cancer prevention [64]. Gallic acid offers antimicrobial and antifungal properties, while tannins support gastrointestinal health and serve as natural preservatives. Moreover, the dietary fiber in pecan shells enhances gut health, regulates blood sugar, lowers cholesterol, and supports satiety, making it ideal for incorporation into nutraceuticals and clean-label functional foods [65]. These health-promoting properties, combined with advances in green extraction methods, position pecan shells as a sustainable and valuable ingredient for the development of future food and pharmaceutical products. Beyond the major bioactive compounds, pecan shells also contain a variety of secondary phenolic compounds that contribute to their overall antioxidant potential and health benefits. Ferulic acid exhibits significant antioxidant and anti-inflammatory properties and is frequently used in dermatological formulations due to its ability to protect skin from oxidative damage [66]. Vanillic acid is known for its radical-scavenging activity and hepatoprotective functions, making it beneficial for liver support [67]. Caffeic acid provides both cardiovascular and antimicrobial benefits, largely through its modulation of oxidative stress and inhibition of microbial growth [41]. Thymol, a monoterpene phenol typically found in essential oils, has demonstrated strong antimicrobial and antifungal activity [46]. Taxifolin (dihydroquercetin) exhibits potent anti-inflammatory and hepatoprotective effects and contributes to vascular and immune health [45]. Syringic acid is recognized for its antioxidant and neuroprotective roles, which may contribute to the maintenance of cognitive health [47]. *p*-Coumaric acid has been reported to reduce lipid peroxidation and may offer anticancer potential by modulating cellular oxidative responses [48]. Finally, protocatechuic acid enhances endogenous antioxidant systems and has shown cardioprotective and anticancer properties by regulating pathways associated with oxidative stress and apoptosis [50]. Processing of functional foods from pecan shells involves drying, grinding, and defatting, followed by solvent extraction (using ethanol, water, or other environmentally friendly solvents) to obtain bioactive compounds. In line with circular economy principles, these bioactives can be sustainably extracted using green methodologies, such as microwave-assisted and ultrasound-assisted extraction, which can be optimized using response surface methodology, as demonstrated in recent studies [68]. The use of green extraction techniques to recover phenolic compounds, particularly ellagic acid, from pecan nut cake was highlighted by Rodrigues et al. and others. Green extraction techniques such as ultrasound-assisted extraction (UAE), microwave-assisted extraction (MAE), and pressurized liquid extraction (PLE) offer environmentally sustainable alternatives to conventional methods by reducing energy usage, solvent consumption, and extraction time [69]. UAE improves extraction efficiency through acoustic cavitation, which enhances cell wall disruption and solvent penetration. Meanwhile, MAE uses rapid internal heating to accelerate mass transfer and shorten processing time while preserving thermolabile compounds [70]. Ultrasound-assisted extraction (UAE) and microwave-assisted extraction (MAE) have been specifically applied to pecan and walnut shells to efficiently recover ellagic acid and other phenolics by enhancing cell wall disruption and accelerating mass transfer, respectively [71]. Additionally, pressurized liquid extraction (PLE) has been used to extract bound phenolic compounds from walnut shells at elevated pressure and temperature, reducing solvent use [72]. Several studies have used similar procedures to recover bioactive chemicals from pecan and walnut shells, with demonstrable improvements reported. For example, UAE of pecan and walnut shells produced ellagic acid concentrations ranging from 18 to 32 mg/g dry weight, depending on sonication time and solvent polarity, with a total phenolic content of up to 120 mg GAE/g [72]. Similarly, MAE has been shown to recover 20–40 mg/g of ellagic acid and other hydrolyzable tannins from nut shells, which represents a 1.5–2.0-fold increase over traditional extraction methods [70]. However, minimal research on green extraction of pecan shells, the complexity of optimization, limited applications for heat-sensitive compounds, and issues related to standardization might hinder industrial use of pecan shell extraction.

This work further emphasizes the role of pecan byproducts in promoting a sustainable and value-added agro-industrial chain, opening new opportunities to use pecan residues in developing pharmaceutical, cosmetic, and nutraceutical products. Given how inexpensive and abundant pecan waste is, funding biopharmaceutical research and development that uses this resource could reduce manufacturing costs while encouraging eco-innovation in public health. Pecan nutshells are a promising low-cost, scalable source of bioactive compounds, and including them in food systems for humans and animals not only enhances nutritional and medicinal benefits but also supports sustainability and waste reduction in agro-industries.

### 3.8. Activated Carbon from Pecan Shells and Its Applications

Activated carbon derived from pecan shells has shown potential as a sustainable and efficient adsorbent for both organic and inorganic pollutants in aqueous environments [7]. Due to their high carbon content, low ash, and naturally porous structure, pecan shells are considered a superior feedstock for producing granular and powdered activated carbon through pyrolysis and activation processes [73]. There is also variability in shell composition across cultivars and origins, affecting standardization. The resulting materials typically exhibit a large specific surface area and a well-developed pore structure, enabling high adsorption capacity.

Regarding inorganic contaminants, pecan shell-based activated carbon has shown strong adsorption abilities for heavy metals such as copper (Cu^2+^), lead (Pb^2+^), and cadmium (Cd^2+^) [74]. These pollutants are common in industrial wastewater, and removing them is vital for protecting aquatic life and human health. Compared to commercial carbons (like bituminous or coconut-based), pecan shell carbons perform equally well or better in metal removal, likely due to increased pore volume and surface functionalities formed during activation. Urban soils often face heavy metal contamination, and this pecan shell carbon could be beneficial for addressing those issues [75].

When targeting organic pollutants, these activated carbons have been used effectively to remove dyes, phenolic compounds, and pesticides from industrial effluents [76]. For example, acid-washed pecan shell carbons showed superior adsorption of methylene blue, a synthetic dye, compared to bituminous-based commercial carbons [73]. The ability to selectively adsorb both low and high-molecular-weight compounds extends their utility to textile, chemical, and pharmaceutical wastewater treatment.

Environmentally, the use of pecan shell-based activated carbon contributes to water purification, soil remediation, and the reduction in nutrient pollution. Their capacity to bind excess nutrients, pesticides, and herbicides supports safer agricultural practices and reduces the risk of groundwater contamination. These features also make them suitable for household water filtration, particularly in rural and underserved communities, thereby supporting affordable access to clean water and enhancing climate resilience. As for emerging contaminants, research on the use of pecan shell-based activated carbon for PFAS (per- and polyfluoroalkyl substances) removal is still limited. However, studies on activated carbon in general show that surface modification and micropore tuning can enhance PFAS adsorption, suggesting a pathway for pecan shell carbons to be adapted for such use [77].

Pecan shell-derived activated carbon offers advantages over conventional carbons in terms of cost, availability, and sustainability. Production costs remain low ($2.72–$2.89/kg), making it competitive with commercial alternatives [7]. Its use reduces biomass waste and supports circular bioeconomy strategies. Technological advances, such as microwave-assisted activation, enzymatic pretreatment, and chemical functionalization, may further improve adsorption efficiency, particularly for complex pollutants. While data on per- and polyfluoroalkyl (PFAS) substances are limited, the high surface area and tunable porosity suggest future potential applicability of pecan shell-based activated carbon [78].

### 3.9. Soil Microbes Improvement from Pecan Shells (Figure 5)

Soil microbes are crucial to the decomposition and nutrient cycling of pecan shells, which, in turn, could contribute to soil health [79]. There are many benefits to the degradation of pecan shells for soil: (1) Soil microbes could decompose the organic matter in pecan shells, thereby mineralizing nutrients. The decomposition process could enhance soil physical properties, such as water holding capacity, nutrient retention, and soil structure [80]. Pecan shells could also increase soil phosphorus content and labile carbon content, which microbes and plants can further utilize. (2) The increased soil aggregation is attributed to microbial decomposition of organic matter and the release of microbial by-products that may bind soil particles. (3) The decomposition of pecan shells can significantly increase the abundance or diversity of beneficial bacteria and fungi. (4) Lastly, pecan shells contain many phenolic compounds, such as gallic acid, which have natural antimicrobial properties and may help manage specific microbial populations [36].

However, the impact of pecan shell soil amendment efficiency depended on soil type, with greater effects on clay loam and sandy loam soils in the short-term treatment, but not on sandy clay [81]. The optimal dosage of pecan shell and pecan shell biochar in soil requires further investigation, since studies on other nut shells indicated a dosage limit for enhancing soil microbes [82]. Disadvantages of using a large amount of pecan shells also exist. Pecan shells also contribute to a reduction in soil-available nitrogen through microbial immobilization, which can lead to plant growth deficiencies if not addressed [83]. Several strategies can be utilized to solve the problems: (1)Through the grinding process, the size of pecan shells can be vastly reduced, which can be more easily applied to an agricultural environment for crops or other plants. (2) Enzymatic treatment of pecan shells: cellulose, hemicellulose, lignin-modifying enzymes, and other enzymes can be used to break down the complex structures of pecan shells before their utilization in soils. (3) NaOH can be used to break down and remove lignin from the pecan shell structure. Through these methods, pecan shells could be efficiently utilized by soil microbes. (4) Microbial enzymatic degradation, such as anaerobic fungi-assisted decomposition processes, also occurs [84].

In addition to soil incorporation, pecan shells have also been used as mulch in horticultural and landscape settings. The application of pecan shells has increased the wet aggregate stability, thereby further enhancing resistance to erosion and improving water infiltration. The direct incorporation of ground pecan shells into arid soils can significantly improve soil structure and biological activity [85]. Their durability and slow decomposition rate make them a long-lasting option for weed suppression and moisture retention. However, minimally processed shells may contain residual kernel fragments, which can attract wildlife, such as squirrels. This characteristic may be a consideration in residential or orchard settings where animal activity is a concern. On the other hand, when properly managed, the potential benefits of pecan shell mulch can be achieved [86]. Expanding pecan shell applications through nanotechnology and building on current findings regarding the antimicrobial and antioxidant activities of pecan shell polyphenols, future research could explore their incorporation into nano-formulations to enhance therapeutic efficacy and stability. Techniques such as nanoparticle encapsulation may improve the delivery and controlled release of bioactive compounds, making them suitable for use in functional foods, pharmaceutical agents, or natural preservatives [87]. This approach, as discussed in recent studies on phenolic compound delivery systems, opens new avenues for valorizing pecan byproducts through nanotechnology-enhanced applications [87].

### 3.10. Pecan Shell Biochar Application in Soil

Pecan byproducts, particularly shells, have been investigated for their potential to enhance soil physical properties, improve water management, and contribute to environmental sustainability [86]. A study conducted on Norfolk loamy sand in South Carolina utilized pecan shell biochar at application rates of up to 44 tons per hectare [88]. The findings indicated that biochar significantly reduced soil penetration resistance, facilitating root development and potentially reducing the need for energy-intensive deep tillage. The biochar exhibited enhancements in water retention, although its effects on soil aggregation and infiltration were inconsistent, possibly influenced by the feedstock and production technique [76,77]. Beyond soil structure improvement, pecan shell biochar offers a range of additional applications: (1) Nutrient retention and cycling: Biochar can adsorb and slowly release essential nutrients such as nitrogen and phosphorus, reducing leaching losses and improving fertilizer efficiency [81]. (2) Carbon sequestration: As a stable form of organic carbon, biochar contributes to long-term carbon storage in soils, thereby playing a role in climate change mitigation [89]. (3) Heavy metal immobilization: Biochar has been shown to immobilize toxic elements such as lead (Pb), cadmium (Cd), and arsenic (As), reducing their bioavailability in contaminated soils [90]. (4) Reduction in greenhouse gas emissions: When incorporated into soil, biochar can lower emissions of nitrous oxide (N_2_O) and methane (CH_4_), particularly in high-input agricultural systems [91]. (5) Microbial habitat enhancement: Its porous structure provides a favorable environment for beneficial microbial communities, enhancing soil biological activity [92]. This highlights the opportunity to optimize water utilization in pecan cultivation, particularly through adjusting irrigation in response to crop load and seasonal needs. Collectively, these studies demonstrate that pecan waste materials, in the form of biochar, compost, or effective water management practices, can enhance soil structure, nutrient accessibility, and water conservation. In a study by Garcia-Perez et al., the potential of pecan shell-derived biochar as a critical material for sustainable urban green infrastructure was emphasized [93]. The review indicates that biochars derived from lignocellulosic feedstocks, such as pecan shells, possess high carbon stability, a significant surface area, and considerable porosity. One promising avenue for urban agriculture is the use of pecan shell biochar to address soil contaminants in historically urban or industrial areas. These characteristics are particularly beneficial for enhancing soil in urban environments. In light of these findings, future research could focus on customizing pecan shell biochar for urban agriculture systems, such as container farming or vertical gardens. Its agronomic efficacy may be enhanced through functional enhancements, such as micronutrient enrichment or inoculation with plant growth-promoting microbes. In addition to promoting circular economy solutions by valorizing agricultural residues, these innovations also improve plant performance and soil health in space-limited urban settings.

## 4. Challenges and Future Directions

Although pecan shells present promising opportunities for conversion into biochar, bioplastics, functional food ingredients, and bioactive extracts, several challenges limit their industrial adoption. Variability in shell composition across cultivars affects extraction efficiency and standardization, while pretreatment and green extraction methods still face scalability constraints, high energy requirements, and inconsistent yields. Economic barriers, including the cost of advanced extraction technologies and limited pilot-scale infrastructure, also impede commercialization relative to competing residues such as walnut and almond shells. Environmental considerations, such as potential wastewater generation during extraction and emissions from thermochemical processes, highlight the need for optimized, low-impact processing strategies. Future efforts should focus on improving preprocessing and separation techniques, developing scalable green extraction pathways, and validating pecan-derived products through techno-economic and life-cycle assessments. Collaboration between industry and research institutions will be essential to advance high-value applications and fully integrate pecan shells into circular bioeconomy models.

## 5. Conclusions

This review analyzed the environmental impact of pecan nutshell waste and its numerous applications in bioenergy, material science, food, nutraceuticals, and medicine. Pecan shells, which contain a high concentration of lignocellulosic and bioactive compounds, have been found to improve soil health, reduce greenhouse gas emissions, and serve as renewable inputs for sustainable energy, biodegradable composites, and antioxidant compositions. Valorization activities that use technologies like pyrolysis, green extraction, and biocomposite processing reduce environmental impact and create economic prospects, especially in rural and agro-industrial areas. Emerging research offers innovations such as biopesticides, activated carbon, antimicrobial compounds, and functional food products. However, challenges remain in scaling production, optimizing extraction techniques, and ensuring consumer safety and regulatory compliance. These methods align with circular production models and low-carbon strategies, which are increasingly important in sustainable agri-food systems. Researchers have demonstrated the increasing use of pecan byproducts and highlighted the need for ongoing research and development to overcome technical obstacles and enhance the financial and environmental benefits of pecan processing. The review highlights that pecan shells are rich in polyphenolic compounds, which possess significant antioxidant and antimicrobial properties, making them promising for applications in the pharmaceutical and food industries. A study conducted by Jéssica Vieira de Souza investigates various extraction methods, including maceration, Soxhlet, and ultrasound-assisted extraction, to isolate these bioactive compounds [94]. It emphasizes the need for further exploration of these compounds in the development of functional foods, natural preservatives, and therapeutic products. Another study conducted by Vieira de Souza underscores the sustainability and efficiency of green extraction methods, particularly ultrasound-assisted extraction in conjunction with ethanol-water solvents, as a sustainable and effective method for the isolation of valuable bioactive compounds from pecan shells [95]. Future initiatives must focus on comprehensive life cycle assessments, techno-economic studies, and multi-stakeholder cooperation to transform pecan biomass from agricultural waste into a strategic resource within circular and sustainable value chains, thereby taking advantage of these opportunities.

## Figures and Tables

**Figure 5:**
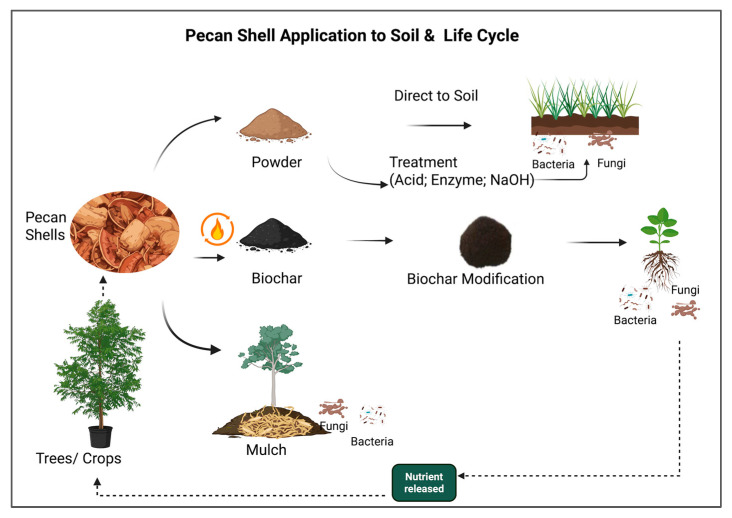
Pecan shell application to soil.

**Table 1:** Screening and selection criteria for study inclusion and exclusion.

Document Inclusion Criteria	Document Exclusion Criteria
Article Published In English	Non-English publications
Peer-reviewed original research articles and reviews	Theses, dissertations, conference abstracts, book chapters, non-peer-reviewed material
Studies published between 2005 and 2025	Studies published before 2005
Studies related to pecan or walnut shell valorization, biochar, activated carbon, extraction of phenolics, or circular bioeconomy	Studies unrelated to nutshell valorization, extraction, or environmental applications
Articles reporting extraction techniques, adsorption properties, physicochemical characterization, bioactive compound yields, or environmental applications	Articles that focus solely on agronomic traits, food chemistry unrelated to shell valorization, or non-material uses

**Table 2:** Bioactive compounds of pecan shell: structures and health-promoting functions.

Bioactive Compounds	Group	Structure	Representative Functions	Typical Extraction Yield (%)	Bioavailability	Industrial Application Potential	Extraction Reference
Benzoic acid	Hydroxybenzoic acids	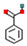	Regulate gut functions	0.2–1.0%	High (well absorbed, rapid metabolism)	Food preservative, antimicrobial agent	[14,31]
Ellagic acid	Hydroxybenzoic acids	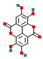	Against oxidation-linked chronic diseases such as cancer and cardiovascular diseases	0.1–0.5%	Low (poor solubility, slow absorption)	Nutraceuticals, anti-aging cosmetics	[32,33]
Gallic acid	Hydroxybenzoic acids	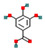	Antioxidant, anticancer, antimicrobial, gastrointestinal, cardiovascular, metabolic, neuropsychological and miscellaneous diseases	1–5%	Moderate–High	Natural antioxidant, pharmaceuticals	[34,35]
p-Hydroxy benzoic acid	Hydroxybenzoic acids	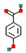	Antioxidant, anti-inflammatory, and intestinal barrier-repairing effects	0.2–1.2%	Moderate	Cosmetic preservatives (parabens precursor)	[33,36]
Protocatechuic acid	Hydroxybenzoic acids	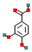	Antioxidant, anti-inflammatory, Neuroprotective properties	0.3–2.0%	Moderate	Anti-inflammatory and neuroprotective ingredients	[33,36]
Pyrogallic acid	Hydroxybenzoic acids	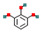	Reducing agent (such as metal), antiseptic properties	0.1–0.3%	Moderate	Hair dyes, inks, metal processing	[32,37]
Vanillic acid	Hydroxybenzoic acids	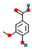	Antioxidant, anti-inflammatory, and anti-cancer	0.2–1.5%	Moderate	Flavoring, fragrance industry	[38]
2-Hydroxycinnamic acid	Hydroxycinnamic acid derivatives	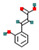	Antioxidant, anti-inflammatory, and antimicrobial agent	0.3–1.8%	Moderate	Food additives, antimicrobial coatings	[39,40]
Chlorogenic acid	Hydroxycinnamic acid derivatives	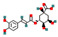	Regulate blood sugar, lipid metabolism, anti-inflammatory, and anti-obesity	0.5–6%	Low-moderate	Anti-obesity supplements, cosmetics	[41]
Ferulic acid	Hydroxycinnamic acid derivatives	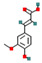	Anti-inflammatory, antimicrobial properties	0.4–2.5%	Moderate	UV-protective cosmetics, food antioxidants	[42,43]
Caffeic acid	Hydroxycinnamic acid derivatives	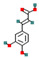	Antioxidant, anti-inflammatory, and anticancer properties	0.4–3.0%	Moderate-High	Natural preservatives, skincare products	[44,45]
Hesperetin	Flavones	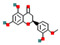	Antioxidant, anti-inflammatory, and neuroprotective properties	0.05–0.3%	Low	Functional beverages, supplements	[46,47]
Kaempferol	Flavonols	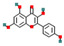	Antioxidant, anti-inflammatory agent	0.1–1.2%	Low (poor water solubility)	Anti-cancer research, nutraceuticals	[48,49]
Quercetin	Flavonols	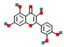	Antioxidant, anti-inflammatory agent	0.2–2.0%	Low–Moderate (improves with glycosides)	Nutraceuticals, anti-allergy formulations	[50,51]
Rutin	Flavonols	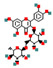	Antioxidant, anti-inflammatory agent	0.1–1.0%	Low	Vascular supplements, pharmaceuticals	[52]
Naringenin	Flavanones	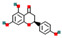	Antioxidant, anti-inflammatory, anti-cancer, anti-diabetic, and neuroprotective effects	0.05–0.4%	Low	Diabetes-related supplements	[53]
Catechin	Monomeric flavan-3-ols	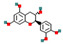	Antioxidant, neuroprotective, anti-cancer	0.3–3.8%	High	Functional beverages, pharmaceuticals	[54]
Epicatechin	Monomeric flavan-3-ols	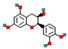	Antioxidant, nitric oxide production, improve blood flow, enhance muscle growth	0.3–4.0%	High	Sports nutrition, cardiovascular supplements	[55]
Epicatechin gallate	Monomeric flavan-3-ols	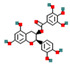	Antioxidant, anti-inflammatory, cardiovascular health, cancer chemoprevention, and neuroprotective properties	0.1–1.2%	Moderate	Cardioprotective formulations	[55]
Epigallocatechin	Monomeric flavan-3-ols	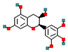	Antioxidant, anti-inflammatory, anti-carcinogenic, neuroprotective, and anti-fibrotic properties	0.2–1.5%	Moderate	Cosmetic antioxidant, nutraceuticals	[56]
Procyanidins	Oligo and polymeric flavan-3-ols	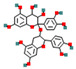	Antioxidant, anti-inflammatory, cardioprotective, anti-cancer, neuroprotective, and metabolic health	1–10% (highest yield class)	Low–Moderate (large molecules)	Cocoa extracts, cardioprotective nutraceuticals	[57]
Procyanidin B1 (B-type dimer)	Oligo and polymeric flavan-3-ols	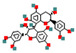	Anti-inflammatory and anti-oxidative processes	0.5–4%	Low	Functional foods, pharmaceuticals	[58]
Procyanidin B2 (B-type dimer)	Oligo and polymeric flavan-3-ols	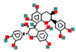	Cardiovascular health, metabolic regulation, wound healing, neuroprotection, reproductive health, anti-inflammatory and antioxidant	0.5–4%	Low	High-value nutraceuticals	[58]

## Data Availability

No new data were generated from this review manuscript.
